# Changes in and the association of retinal blood perfusion and retinal nerves in diabetic patients without retinopathy

**DOI:** 10.3389/fendo.2022.1036735

**Published:** 2023-01-17

**Authors:** Jianchen Hao, Jiantong Du, Xiaopeng Gu, Yadi Zhang, Liu Yang, Shijie Zhang

**Affiliations:** Department of Ophthalmology, Peking University First Hospital, Beijing, China

**Keywords:** ganglion cell-inner plexiform layer, diabetic retinopathy, intraretinal blood flow perfusion, GCIPL thickness, RNFL thickness

## Abstract

**Objective:**

To explore intraretinal blood flow perfusion and nerve changes, as well as the correlation between them, in diabetic patients without diabetic retinopathy (NDR).

**Method:**

Eighty-six NDR patients (86 eyes) who attended the ophthalmology clinic between December 2019 and December 2021 were included. Sixty-four eyes of 64 healthy examined controls in the same period were selected as the control group. The patients underwent routine ophthalmological examination, optical coherence tomography (OCT) and OCT angiography.

**Results:**

The average thickness, minimum thickness and thickness of each quadrant except for the superior temporal quadrant of the ganglion cell-inner plexiform layer (GCIPL) in the macular area of the affected eyes in the NDR group were lower than that of the tested eyes in the control group (*P* < 0.05). The average retinal nerve fibre layer (RNFL) thickness of the NDR group and the superior, inferior and nasal quadrants around the optic disc of the affected eyes in the NDR group were lower compared with the tested eyes in the control group (*P* < 0.001, *P =* 0.003, *P* = 0.001, *P* = 0.009). The mean vessel length density in the parafoveal and perifoveal areas in the NDR group was positively associated with the mean GCIPL thickness in the macular area (*ρ* = 0.265, *ρ* = 0.257 and *P* < 0.001). No blood flow perfusion parameters in the NDR group were correlated with the RNFL thickness of the corresponding quadrant around the optic disc (*P* > 0.05).

**Conclusion:**

In diabetic patients without diabetic retinopathy, the superficial retinal vessel density in the macular area positively correlated with GCIPL thickness, and the superficial retinal vessel density around the optic disc was not correlated with RNFL thickness.

## 1 Introduction

Diabetic retinopathy (DR) is a series of lesions caused by retinal microvascular damage due to diabetes (1). It is a chronic and progressive disease that leads to vision loss or even blindness, which may place a heavy economic burden on society (2–4). Studies have confirmed that regular follow-up and effective intervention in DR patients could avoid severe vision loss in 90% of patients (5). Therefore, early diagnosis, regular monitoring and the early treatment of DR are critical for delaying the development of lesions and reducing visual loss (6).

The diagnosis of DR requires an ophthalmologist to evaluate the fundus examination in diabetic patients, which sometimes requires fundus fluorescein angiography (FFA) and optical coherence tomography (OCT) (7); however, these two modalities both have limitations (8). For this reason, OCT angiography (OCTA) has become an important imaging examination for the diagnosis, follow-up and study of the pathogenesis of various fundus diseases, including DR (9, 10).

Recent studies suggested that diabetic retinal neurodegeneration may predate DR (11). Optical coherence tomography measurement of retinal-related structural parameters, including retinal ganglion cell (RGC)-related parameters in the macular area and retinal nerve fibre layer (RNFL) thickness, could provide a reference for evaluating the damage of retinal nerve cells (12). In recent years, studies have also indicated that in patients with diabetes mellitus without diabetic retinopathy (NDR), OCTA and OCT can be used to observe retinal blood flow perfusion and retinal nerve changes before DR appears (13–15). However, there remains uncertainty about the microvascular and neurological injuries that could be induced by diabetes mellitus at an early stage, particularly for NDR patients. Future research in this field may be able to reveal the underlying rules and provide evidence for the early screening of preclinical diabetic retinopathy.

The current study aimed to apply OCTA to observe blood perfusion changes in the macular area and around the optic disc in patients with NDR and to observe the thickness of the ganglion cell-inner plexiform layer (GCIPL) in the macular area and RNFL around the optic disc.

## 2 Methods

### 2.1 Study design

This was a randomised controlled study. With a random card, 86 eyes of 86 patients with type 2 diabetes without DR, who had visited the ophthalmology clinic of Peking University First Hospital between December 2019 and December 2021 were selected (NDR group), and 64 eyes from 64 health examination cases in the same period were selected as the control group. Both patients and healthy individuals were assigned a random number, some of which identified them as participating in the study while other numbers represented non-participating patients. This selection method helps to ensure the random nature of participant screening. Patients who took a random number signifying participation in the study moved on to the next process for further screening according to the inclusion and exclusion criteria. The researchers completed the randomised assignment of the participants.

The right eye of all subjects was selected for inclusion. The left eye was selected for inclusion only when the right eye’s OCTA image quality was substandard. The Ethics Committee of Peking University First Hospital approved this study, and all participants signed an informed consent form before enrolling.

The inclusion criteria for the diabetic patients without diabetic retinopathy were as follows: (1) Patients met the diagnostic criteria for type 2 diabetes mellitus (16) and (2) did not meet the DR diagnostic criteria in both eyes as established *via* a fundus examination using slit-lamp microscopy, an additional non-contact lens and fundus photograph examination after mydriasis (7).

The inclusion criteria for the control group were as follows: (1) Patients had no history of diabetes mellitus and (2) no history of ocular diseases other than mild or moderate ametropia.

The exclusion criteria for the NDR and the control groups were: (1) retinal and choroidal diseases, such as retinal vascular obstructive disease, macular oedema, epiretinal membrane, macular hole, age-related macular degeneration and uveitis; (2) intraocular pressure >21 mmHg (1 mmHg = 0.133 kPa), glaucoma, suspicious glaucoma, ocular hypertension or other optic nerve diseases; (3) dioptre > +5.0 D or < –6.0 D; (4) axial length >26 mm or <21 mm; (5) the ocular media opacity affected the image quality of the fundus examination or OCTA and OCT examinations; (6) a history of intraocular surgery except for a history of phacoemulsification surgery for more than one year, such as intravitreal drug injection, retinal photocoagulation, vitreoretinal surgery and glaucoma surgery; (7) a history of ocular trauma; (8) neurological diseases, such as Alzheimer’s and Parkinson’s disease and multiple sclerosis; (9) systemic diseases, such as serious cardiovascular diseases, blood diseases and rheumatic and immune diseases; (10) pregnancy; (11) the image obtained by multiple OCTA and (or) OCT examination in both eyes was unqualified; (12) HbA1c > 7%, diabetic nephropathy or a confirmed diagnosis of diabetes mellitus (fasting blood glucose ≥ 7.0 mmol/L).

### 2.2 Routine ophthalmic examination

All subjects underwent routine ophthalmic examinations, including: (1) best corrected visual acuity (BCVA) using the international standard visual acuity table (the statistics were converted to the logarithm of the minimum angle of resolution [logMAR] visual acuity); (2) intraocular pressure using the non-contact tonometer CT-60 (Topcon Company, Japan); (3) anterior segment examination using a slit-lamp biological microscope (Haag–Streit, Switzerland, the same below); (4) axial length measurement using the optical coherence interferometry IOLmaster500 (Carl Zeiss, Germany); (5) fundus examination using a slit-lamp biological microscope and additional 90D non-contact lens after mydriasis with eye drops comprising 0.5% tropicamide mixed with 0.5% phenylephrine hydrochloride (Volk, USA); (6) fundus photography after mydriasis using a TRC-DX50 fundus camera (Topcon Company, Japan).

### 2.3 Optical coherence tomography angiography examination

Subjects were examined for OCTA using a Cirrus HD-OCT 5000 (American Carl Zeiss, Inc.). The same skilled inspector performed the inspection. The same film reader confirmed the accuracy of the automated retinal stratification before conducting image parameter measurements. Signal intensity was quantitatively assessed from 1 (poor) to 10 (good). When one or more of the following conditions occurred, the assessed image was unqualified: signal intensity <7, ocular media opacity or an artefact caused by vitreous floating objects, poor fixation coordination or an artefact caused by scardamyxis.

**Figure 1:**
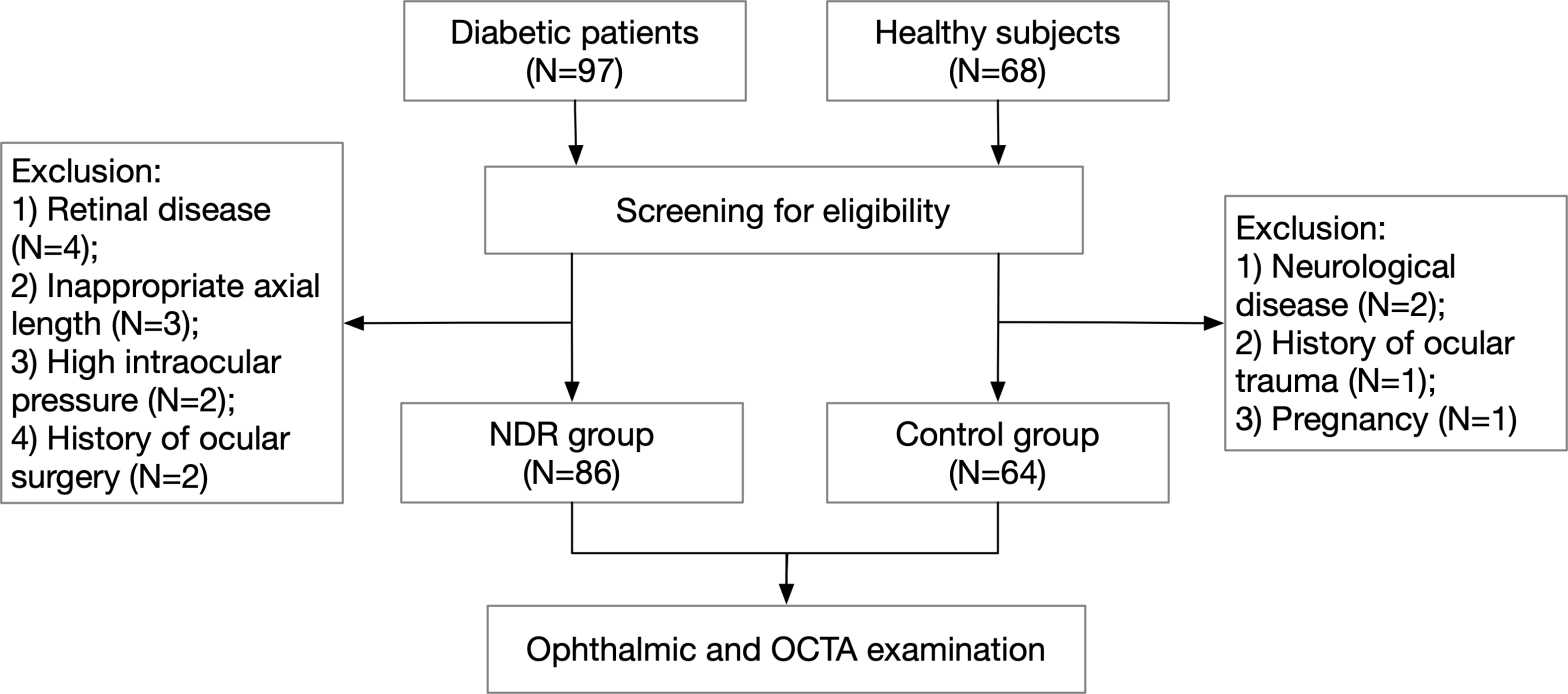
Flow diagram of patient selection. NDR, non-diabetic retinopathy; OCTA, optical coherence tomography angiography.

**Figure 2:**
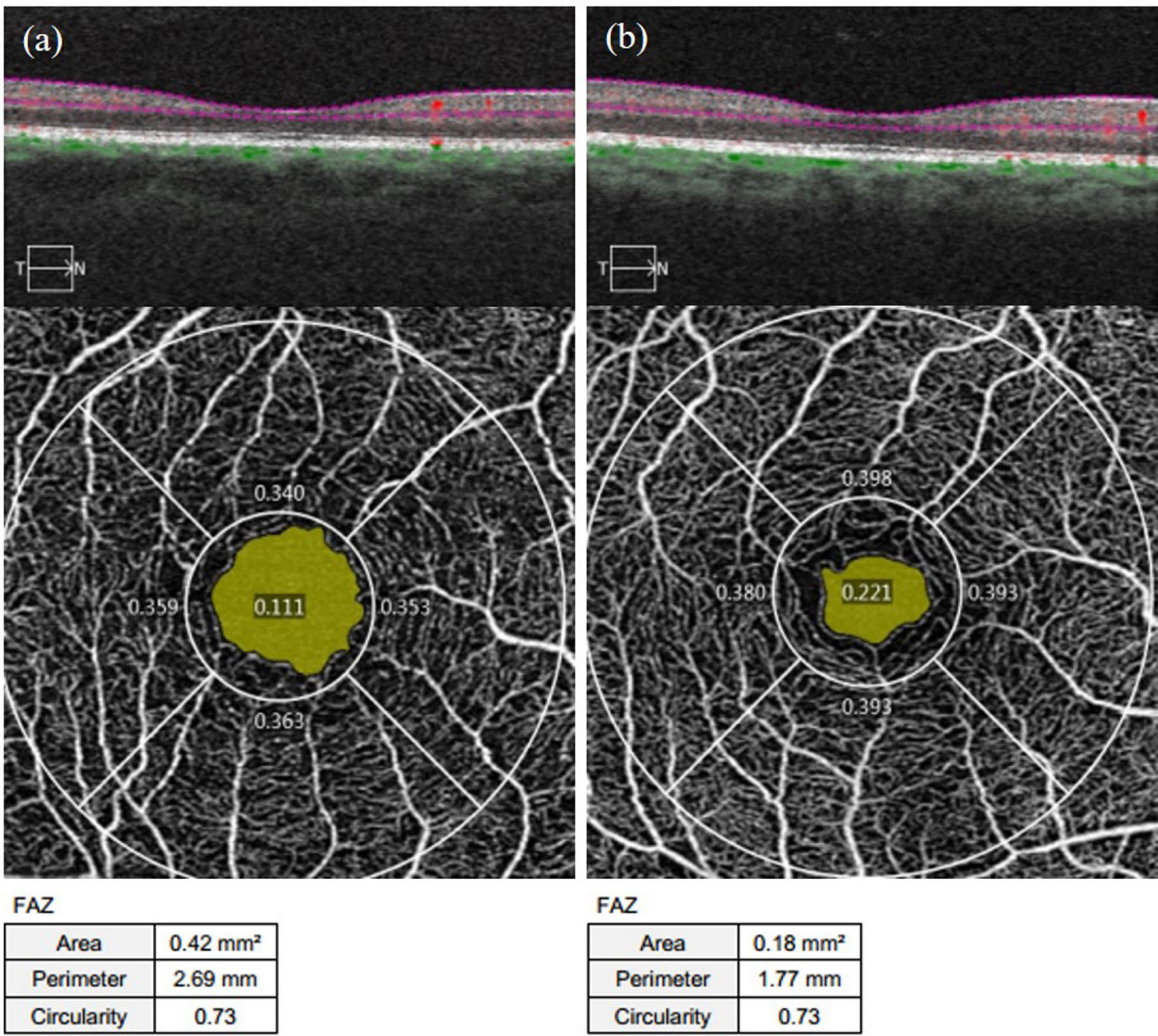
Representative OCTA images of the FAZ area, perimeter, and circularity measurements of the affected eyes in the NDR group and the tested eyes of the control group. **(A)** results of 3 mm×3 mm B scan of the OCTA in the macular area, superficial retinal en face images, and FAZ area, perimeter, and circularity that are automatically measured by the system software in the right eye of a 63-year-old female NDR patient. **(B)** results of 3 mm×3 mm B scan of the OCTA in the macular area, superficial retinal en face images, and FAZ area, perimeter, and circularity that are automatically measured by the system software in the right eye of a 54-year-old female control. The yellow area shows the FAZ area automatically identified by the system software. The FAZ area and perimeter of the affected eyes of the NDR patients were larger compared to the eyes of the control, and the FAZ circularity was equal. NDR, diabetes mellitus without diabetic retinopathy; FAZ, foveal avascular zone; OCTA, optical coherence tomography angiography.

**Figure 3:**
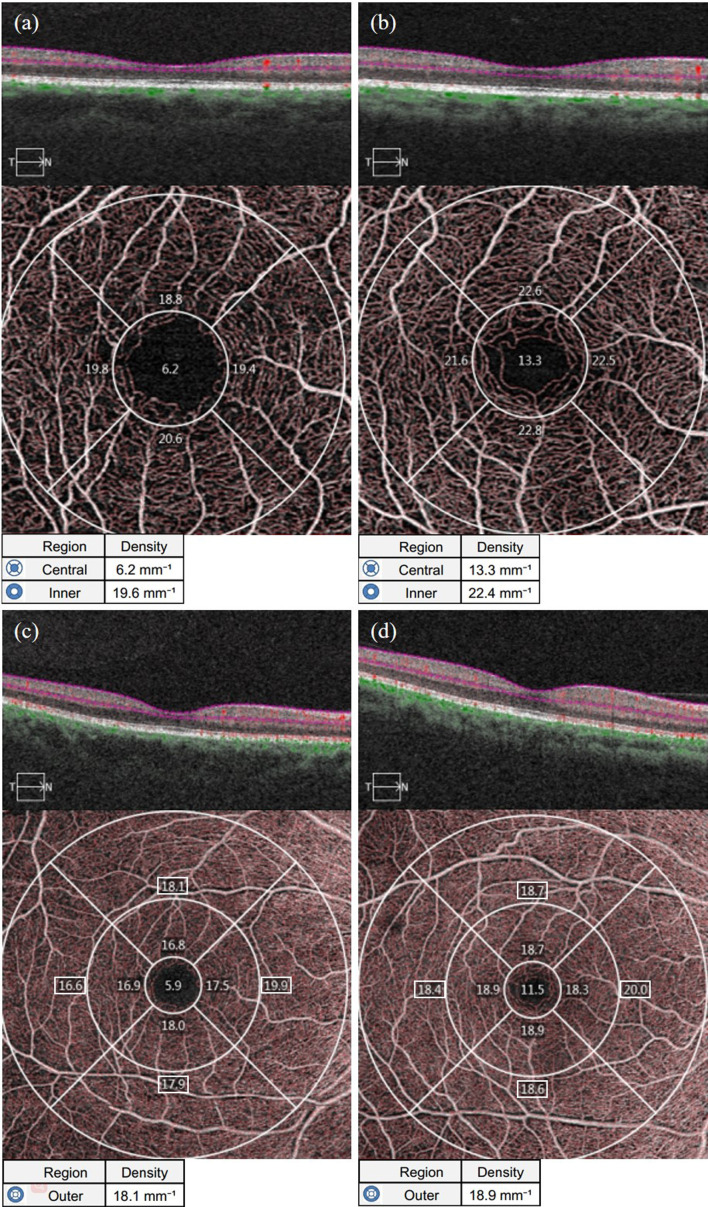
Representative OCTA images of VLD measurements in the macular area of the affected eyes in the NDR group and the tested eyes in the control group. **(A)** Results of OCTA macular area 3 mm×3 mm B scan, the en face image of the superficial retina and VLD of the foveal area (central item in the table, the same below), mean VLD of the parafoveal area (inner item in the table, the same below), and VLD in each quadrant of the parafoveal area (The corresponding quadrant number in the figure, the same below) automatically measured by the system software of the right eye of a 63-year-old female NDR patient. **(B)** Results of OCTA macular area 3 mm×3 mm B scan, the en face image of the superficial retina and VLD of the foveal area, mean VLD of the parafoveal area, and VLD in each quadrant automatically measured by the system software of the right eye of a 54-year-old female control. **(C)** Results of OCTA macular area 6 mm×6 mm B scan, the en face image of the superficial retina and VLD of the foveal area (outer item in the table, the same below), mean VLD of the parafoveal area, and VLD in each quadrant of the parafoveal area (The corresponding region number in the figure, the same below) automatically measured by the system software of the right eye of NDR patients as in **(A)**. **(D)** Results of OCTA macular area 6 mm×6 mm B scan, the en face image of the superficial retina and mean VLD of the parafoveal area, and VLD in each quadrant of the parafoveal area automatically measured by the system software of the right eye of the control as in **(B)**. The VLD results in the macular area of the NDR patients were smaller than those of the normal control. NDR, diabetes mellitus without diabetic retinopathy; VLD, vessel length density; OCTA, optical coherence tomography angiography.

As shown in **Figures 1–3 of the Appendix**, the angiography-scanning mode was selected with the macular region or the optic disc region as the scanning site. The scan range was 3 × 3 mm or 6 × 6 mm with a scan depth of 2 mm. Blood perfusion parameters of the superficial retina were measured using AngioPlex software (v.10.0). The macular blood perfusion measurement areas were the foveal avascular zone (FAZ), foveal area, parafoveal area and the perifoveal area. Blood perfusion parameters included the FAZ area, FAZ perimeter, FAZ circularity (4πA/P^2^, where A is the FAZ area, and P is the FAZ perimeter), vessel length density (VLD, defined as the total structured length of blood-flow signal in the unit area), perfusion density (PD, defined as the proportion of the area of blood-flow signal per unit area). The blood perfusion measurement area around the optic disc was focused on the centre of the optic disc, the annular area between two concentric circles of 3 and 6 mm in diameter, which was further divided into four quadrants, i.e. the upper, temporal, inferior and nasal sides. The blood perfusion parameters around the optic disc were the mean and actual VLD and PD in the superior, temporal, inferior and nasal quadrants around the optic disc.

### 2.4 Optical coherence tomography examination

The patients were examined using OCT with a Cirrus HD-OCT 5000 (American Carl Zeiss Company). The same skilled inspector performed the inspection. The signal intensity was quantitatively assessed from 1 (poor) to 10 (good). When the signal intensity was <7, the assessed image was unqualified.

**Figure 4:**
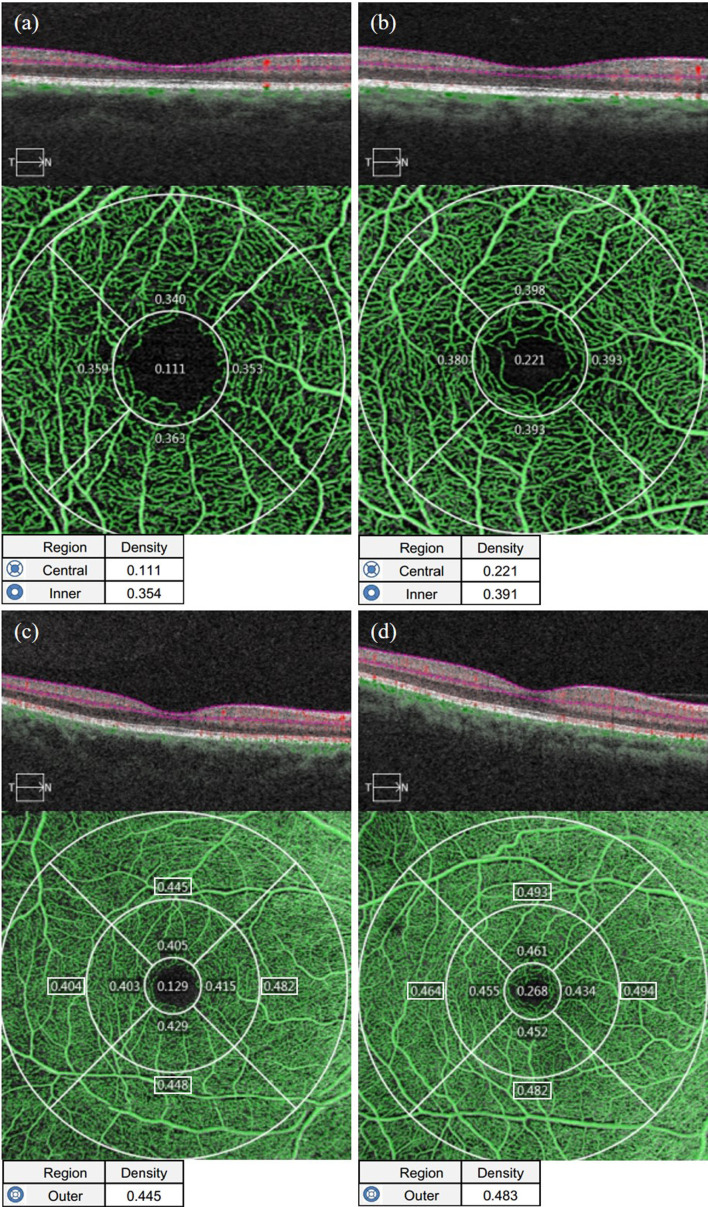
Representative OCTA images of PD measurements in the macular area of the affected eyes in the NDR group and the tested eyes in the control group. **(A)** Results of OCTA macular area 3 mm×3 mm B scan, the en face image of the superficial retina and PD of the foveal area (central item in the table, the same below), mean PD of the parafoveal area (inner item in the table, the same below), and PD in each quadrant of the parafoveal area (The corresponding region number in the figure, the same below) automatically measured by the system software of the right eye of a 63-year-old female NDR patient. **(B)** Results of OCTA macular area 3 mm×3 mm B scan, the en face image of the superficial retina and PD of the foveal area, mean PD of the parafoveal area, and PD in each quadrant automatically measured by the system software of the right eye of a 54-year-old female control. **(C)** Results of OCTA macular area 6 mm×6 mm B scan, the en face image of the superficial retina and PD of the foveal area (outer item in the table, the same below), mean PD of the parafoveal area, and PD in each quadrant of the parafoveal area (The corresponding region number in the figure, the same below) automatically measured by the system software of the right eye of NDR patients as in **(A)**. **(D)** Results of OCTA macular area 6 mm×6 mm B scan, the en face image of the superficial retina and mean PD of the parafoveal area, and PD in each quadrant of the parafoveal area automatically measured by the system software of the right eye of the control as in **(B)**. The PD results in the macular area of the NDR patients were smaller than those of the control. NDR, diabetes mellitus without diabetic retinopathy; PD, perfusion density; OCTA, optical coherence tomography angiography.

**Figure 5:**
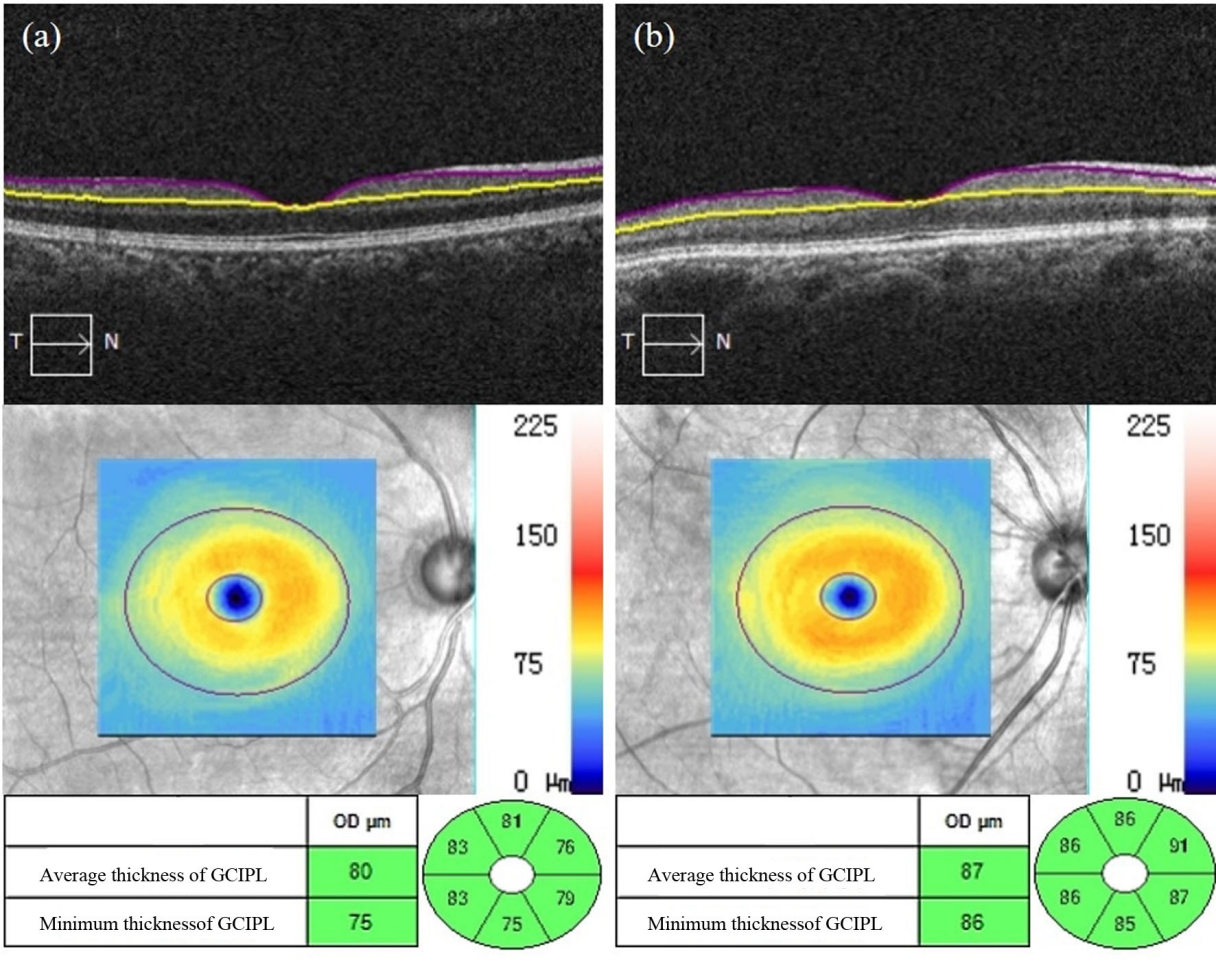
Representative OCT images of GCIPL thickness measurements in the macular area of the affected eyes in the NDR group and the tested eyes in the control group. **(A)** Results of the OCT macular area B scan, GCIPL thickness figure and GCIPL mean, minimum, and each quadrant thickness automatically measured by the system software of the right eye of a 50-year-old male NDR patient. **(B)** Results of the OCT macular area B scan, GCIPL thickness figure and GCIPL mean, minimum, and each quadrant thickness automatically measured by the system software of the right eye of a 67-year-old female control.

As shown in **Figures 4, 5 in the Appendix**, the three-dimensional cubic 512 × 128 scan mode was selected for the macular or optic disc area. The scan range was 6 × 6 mm with a scan depth of 2 mm. The following GCIPL thickness parameters in the macular area were measured: mean thickness, minimum thickness and GCIPL thickness in six quadrants, i.e. the upper, superior temporal, inferior temporal, inferior, nasal inferior and nasal superior quadrants. The following RNFL thickness parameters around the optic disc were measured: the average and actual RNFL thickness in four quadrants (superior, temporal, inferior and nasal sides).

### 2.5 Statistical analysis

Statistical analysis was performed using the SPSS Statistics software (v.23.0; American IBM SPSS Corporation). The data were first tested for normality and homogeneity of variance. Normally distributed measurement data were represented by *x* ± *s*, and an independent sample t-test was used to compare the two groups. The course of diabetes of the non-normally distributed measurement data was expressed by M (range), and the rest was represented by M (P25, P75). The Mann–Whitney U test was used to compare the two groups. A comparison between two groups of two categorical variables was performed using the 2-test. The correlation analysis of measurement data of two normal distributions was performed using Pearson correlation analysis. Spearman’s correlation analysis was used for the measurement data that did not conform to the normal distribution, and *P* < 0.05 was considered a statistically significant difference.

## 3 Results

### 3.1 Basic characteristics of the subjects

This study screened 97 diabetic patients and 68 healthy subjects, finally including a total of 86 eyes (69 right eyes and 17 left eyes) among 86 cases in the NDR group and 64 eyes (50 right eyes and 14 left eyes) among 64 cases in the control group. A flow diagram representing ***** is shown in **Figure 1**. The NDR group comprised 47 men and 39 women. The median duration of diabetes was 10.8 years (4 months to 28 years). The control group included 29 males and 35 females. The differences in gender (*P* = 0.258), age (59.8 ± 9.8 vs. 61.8 ± 7.7, *P* = 0.184), logMAR BCVA (0.023 ± 0.074 vs. 0.006 ± 0.081, *P* = 0.163) and ocular axis length (23.51 ± 0.91 mm vs. 23.43 ± 0.98 mm, *P* = 0.604) in the NDR group were not significant (see **Appendix, Table 1**).

### 3.2 Optical coherence tomography angiography measurement parameters

#### 3.2.1 Foveal avascular zone area, perimeter and circularity

As shown in **Table 2 in the Appendix**, the FAZ area and perimeter of the affected eyes in the NDR group were larger than those of the control group, with statistically significant differences (*P* = 0.011, *P* = 0.001). There was no significant difference in the FAZ circularity of the examined eyes between the two groups (*P* = 0.464). Representative OCTA images are shown in **Figure 2**.

#### 3.2.2 Vessel length density of the macular area

As shown in **Table 1**, the average VLD in the parafoveal area and the area around the fovea in the NDR group was lower than in the control group (*P* = 0.003, *P* = 0.026). The VLD in the upper, temporal and nasal quadrants of the parafoveal area and the upper quadrant of the peripheral foveal area in the NDR group were also lower than in the control group (*P* < 0.01). There was no significant difference in VLD between the two groups in the lower quadrant of the fovea, parafovea and the temporal, inferior and nasal quadrants around the fovea (*P* > 0.05). A representative OCTA image is shown in **Figure 3**.

**Table 1:** VLD results in the macular area of the affected eyes of NDR patients and tested eyes of the control group.

Macular area VLD (mm^-1^)		The NDR group (n=86)	The control group (n=64)	*t*/*Z* value	*P* value
Foveal area		8.19 ± 4.06	9.19 ± 2.79	-1.681	0.095
Parafoveal area	Mean	17.92 ± 1.67	18.44 ± 0.97	-3.025	0.003
	Superior	18.00 (17.28, 18.43)	18.70 (17.92, 19.28)	-3.758	<0.001
	Temporal	17.22 (16.68, 18.10)	18.05 (17.24, 18.50)	-3.234	0.001
	Inferior	18.10 (17.28, 19.12)	18.65 (17.70, 19.18)	-1.918	0.055
	Nasal	19.25 (18.48, 19.90)	19.60 (19.10, 20.10)	-2.090	0.037
Foveal areaPeripheral area	Mean	17.75 (17.08, 18.50)	18.70 (18.10, 19.20)	-2.226	0.026
	Superior	17.64 (16.88, 18.63)	18.30 (17.50, 19.20)	-2.754	0.006
	Temporal	18.00 (17.18, 18.00)	18.45 (17.55, 19.08)	-1.683	0.092
	Inferior	17.90 (17.18, 18.73)	18.45 (17.28, 18.90)	-1.477	0.140
	Nasal	17.75 (16.68, 18.53)	18.20 (17.50, 18.85)	-1.553	0.120

Normally distributed measurement data are represented by x ± s, and independent sample t-test was used for the comparison between the two groups. Non-normally distributed measurement data are represented by M (P25, P75), and the Mann-Whitney U test was used for the comparison between the two groups. NDR, Diabetes mellitus without diabetic retinopathy; VLD, vessel length density.

#### 3.2.3 Perfusion density of macular

As shown in **Table 2**, the average PD in the foveal area and the area near the fovea in the NDR group was lower than in the control group (*P* = 0.005, *P* = 0.003). The PD in the upper and temporal quadrants of the parafoveal area and the upper quadrant of the perifoveal area in the NDR group were also lower than in the control group (*P* < 0.001, *P* = 0.029, *P* = 0.004). There was no significant difference between the two groups in the average parafoveal area, the PD of the inferior and nasal quadrants of the parafoveal area or the PD of the temporal, inferior and nasal quadrants of the peripheral foveal area (*P* > 0.05). A representative OCTA image is shown in **Figure 4**.

**Table 2:** PD results in the macular area of the affected eyes of NDR patients and tested eyes of the control group.

Macular area PD		The NDR group (n=86)	The control group (n=64)	*t*/*Z* value	*P* value
Foveal area		0.1746 ± 0.0750	0.2094 ± 0.0684	-2.828	0.005
Parafoveal area	Mean	0.4560 (0.4368, 0.4703)	0.4705 (0.4510, 0.4810)	-1.765	0.077
	Superior	0.4559 (0.4365, 0.4720)	0.4795 (0.4570, 0.4870)	-4.167	<0.001
	Temporal	0.4345 (0.4050, 0.4550)	0.4500 (0.4286, 0.4625)	-2.185	0.029
	Inferior	0.4595 (0.4380, 0.4813)	0.4740 (0.4420, 0.4870)	-1.657	0.097
	Nasal	0.4790 (0.4600, 0.4923)	0.4910 (0.4698, 0.4968)	-1.893	0.058
Foveal areaPeripheral area	Mean	0.4291 (0.4110, 0.4490)	0.4440 (0.4160, 0.4590)	-2.935	0.003
	Superior	0.4307 (0.4145, 0.4528)	0.4540 (0.4293, 0.4670)	-2.860	0.004
	Temporal	0.4360 (0.4178, 0.4528)	0.4445 (0.4165, 0.4603)	-1.148	0.251
	Inferior	0.4340 (0.4225, 0.4600)	0.4425 (0.4220, 0.4620)	-0.925	0.355
	Nasal	0.4285 (0.3995, 0.4553)	0.4325 (0.4128, 0.4495)	-0.513	0.608

Normally distributed measurement data are represented by x ± s, and independent sample t-test was used for the comparison between the two groups. Non-normally distributed measurement data are represented by M (P25, P75), and the Mann-Whitney U test was used for the comparison between the two groups. NDR, Diabetes mellitus without diabetic retinopathy; VLD, vessel length density.

#### 3.2.4 Vessel length density around the optic disc

**Figure 6:**
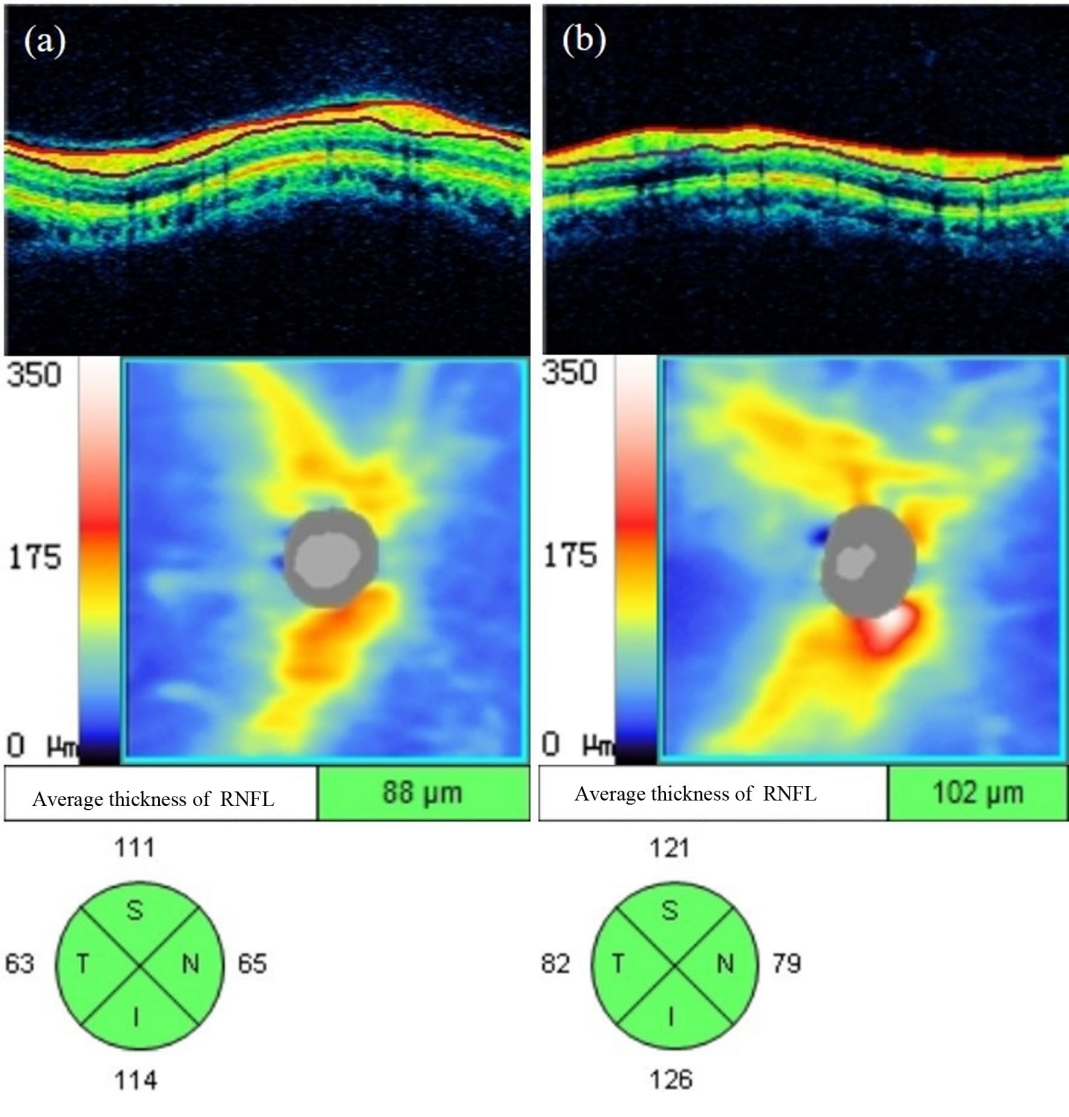
Representative OCT images of RNFL thickness measurements around the optic disc of the affected eyes in the NDR group and the tested eyes in the control group. **(A)** Results of OCT annular ring B scan, RNFL thickness figure, and mean and each quadrant RNFL thickness around the optic disc automatically measured by the system software of the right eye of a 63-year-old male NDR patient. **(B)** Results of OCT annular ring B scan, RNFL thickness figure, and mean RNFL thickness and RNFL thickness in each quadrant around the optic disc automatically measured by the system software of the right eye of a 54-year-old female control. The average RNFL thickness around the optic disc and each quadrant thickness of the NDR affected eyes were smaller compared with the examined eyes of the control. NDR, diabetes mellitus without diabetic retinopathy; RNFL, retinal nerve fiber layer; OCT, optical coherence tomography.

As shown in **Table 3 in the Appendix**, the average VLD around the optic disc and the VLD in the NDR group’s upper, temporal, lower and nasal quadrants of the affected eyes were significantly lower than in the control group (*P* < 0.001). Representative OCTA images are shown in **Figure 6 in the Appendix**.

#### 3.2.5 Perfusion density around the optic disc

As shown in **Table 4 in the Appendix**, the average PD around the optic disc and the PD in the NDR group’s upper, temporal, lower and nasal quadrants of the affected eyes were significantly lower than those of the control group (*P* < 0.05). Representative OCTA images are shown in **Figure 7 in the Appendix**.

### 3.3 Optical coherence tomography measurement parameters

#### 3.3.1 Ganglion cell-inner plexiform layer thickness in the macular area

As shown in **Table 5 in the Appendix**, the average thickness, minimum thickness and thickness of each quadrant, except the superior temporal quadrant of the GCIPL in the macular area of the affected eyes in the NDR group, were lower than that of the tested eyes in the control group and reflected a statistically significant difference (*P* < 0.05). There was no significant difference in GCIPL thickness in the superior temporal quadrant in the macular area between the two groups (*P* = 0.113). Representative OCTA images are shown in **Figure 5**.

#### 3.3.2 Retinal nerve-fibre layer thickness around the optic disc

As shown in **Table 6 in the Appendix**, the average RNFL thickness around the optic disc and the RNFL thickness in the upper, lower and nasal quadrants around the optic disc in the NDR group were significantly lower than in the control group (*P* < 0.001, *P* = 0.003, *P* = 0.001, *P* = 0.009). There was no significant difference in RNFL thickness between the two groups in the temporal quadrant around the optic disc (*P* > 0.05). A representative OCTA image is shown in **Figure 6**.

### 3.4 Correlation between optical coherence tomography angiography measurement parameters and optical coherence tomography measurement parameters

In the NDR group, the average VLD in the parafoveal area, the average VLD in the perifoveal area, the average PD in the parafoveal area and the average PD in the perifoveal area were all positively correlated with the average thickness of the GCIPL in the macular area (**Table 3**). There was no correlation between the FAZ area, circumference, circularity and the mean thickness of the GCIPL in the macular area in the NDR group (*P* > 0.05).

**Table 3:** Correlation between blood perfusion parameters and the mean GCIPL thickness in the macular area of affected eyes in the NDR group.

Blood flow perfusion parameters in the macular area		Mean GCIPL thickness in the macular area	
		correlation coefficient	*P* value
FAZ	Area	*ρ*=-0.59	0.590
	perimeter	*ρ*=-0.072	0.507
	Circularity	*ρ*=-0.049	0.656
VLD	Mean in the parafoveal area	*r*=0.265	0.014
	Mean in the perifoveal area	*ρ*=0.257	0.017
PD	Mean in the parafoveal area	*ρ*=0.427	<0.001
	Mean in the perifoveal area	*ρ*=0.263	0.014

Pearson correlation analysis for measurement data both fitting the normal distribution. The Spearman correlation analysis is used if the two measurement data do not fully meet the normal distribution; n=86, NDR, diabetes without diabetic retinopathy; OCTA, optical coherence tomography angiography; GCIPL, ganglion cell-inner plexiform layer; FAZ, foveal avascular area; VLD, vessel length density; PD, perfusion density; r, Pearson correlation coefficient; ρ, Spearman correlation coefficient.

As shown in **Table 7 in the Appendix**, no blood perfusion parameters (including average VLD, PD, VLD and PD in the upper, temporal, lower and nasal quadrants) around the optic disc in the NDR group correlated with RNFL thickness in the corresponding quadrants around the optic disc (all *P* > 0.05).

## 4 Discussion

This study found that the FAZ area and perimeter of the macular area for the superficial retina expanded, and the blood flow density decreased in the macular area and around the optic disc in NDR patients. The GCIPL in the macular region and RNFL around the optic disc thinned. Moreover, in the macular area, the superficial retinal blood flow density was positively correlated with the GCIPL thickness in NDR patients, and around the optic disc, the superficial retinal blood flow density positively correlated with the RNFL thickness.

In this study, OCT was applied to observe the GCIPL thickness changes in each quadrant of the macular area of NDR patients. The authors found that, compared with the control subjects, the GCIPL thickness was significantly reduced in all quadrants, except for the superior temporal quadrant among NDR patients. In contrast, the thickness of the superior temporal quadrant was not statistically significant. The results of existing studies on GCIPL thickness changes in each quadrant of the macular areas of NDR patients were not completely consistent. However, none of the studies, except for Carpineto (17), found a significant reduction in the superior temporal quadrant thickness (18–20); therefore, the results of this study were generally consistent with the results of existing studies. This may have been because the macular area’s nasal region is part of the macular bundle’s walking region, with a dense distribution of RGCs. However, the distribution of RGCs on the temporal side of the macular area was less dense than on the nasal side, which may indicate that a reduction of GCIPL thickness on the temporal side of the macular area was less significant than on the nasal side (21).

In this study, the thickness of the RNFL in the inferior and nasal quadrants around the optic disc in NDR patients was significantly lower than in the control group. Nonetheless, there was no significant difference between the two groups in the superior and temporal quadrants, which was consistent with selected existing studies (22, 23). It may have been that the RNFL in the temporal quadrant around the optic disc was thinnest compared with the remaining quadrants, leading to a less significant reduction in the RNFL thickness in the temporal quadrant.

Existing OCTA and OCT studies among normal participants found that pan-retinal blood flow density in the parafoveal region of the macular area was positively correlated with inner retinal thickness and not correlated with pan-retinal thickness (24). In existing OCTA studies, adaptive optical scanning laser ophthalmoscope and OCT in normal patients found that the FAZ area negatively correlated with the inner retinal thickness in the foveal area, suggesting that the thicker inner retina in the macular area required a smaller FAZ to meet its metabolic needs (25). Other OCTA and OCT studies on glaucoma showed that the FAZ area was negatively associated with the GCIPL thickness in the macular area, FAZ circularity positively correlated with the GCIPL thickness in the macular region, superficial retinal blood flow density in the macular area positively correlated with the GCIPL thickness in the macular area and pan-retinal flow density in the macular area positively correlated with pan-retinal and inner retinal thickness in the macular area (26, 27). In the present study, the mean VLD and PD in the superficial retina in the perifoveal region of NDR patients positively correlated with the mean GCIPL thickness in the macular region.

Notably, recent studies suggested that diabetes-related abnormal retinal changes resulted from retinal neurovascular unit injuries (28). The composition of retinal neurovascular unit units includes retinal neurons, glial cells (Müller cells, astrocytes, microglia), vascular endothelial cells and pericytes (29). Common mechanisms exist between retinal microcirculation injury and nerve cell injuries in diabetes, such as oxidative stress injury, the excitotoxic effects of glutamate and the unbalanced production of neuroprotective factors (30, 31). In the current study, the authors demonstrated the correlation between retinal blood flow perfusion and retinal nerve changes in NDR patients, which can be partially explained by the above reasons. This phenomenon is of great clinical importance for evaluating microvascular and neurological injuries in diabetic patients at an early stage. Optical coherence tomography angiography may be a promising tool for screening vulnerable patients from diabetes-induced retinopathy. In this way, risk stratification can be improved and decision-making concerning this clinical scenario can be facilitated *via* the implementation of a high-resolution imaging modality, which may help to improve the quality of life and reduce the loss of function among this subset of patients.

This study includes some limitations. First, the sample size of the study was small, and the authors did not dynamically observe the parameters of retinal blood perfusion and retinal nerve changes at different time points among NDR patients. There remains uncertainty about the RNFL thickness changes in each quadrant around the optic disc in NDR patients. In the future, large-sample, longitudinal studies are needed to further confirm the results of this study. Second, this study did not involve measuring deep retinal-related blood-perfusion parameters. However, considering the impact of projection artefacts on the accuracy of the quantitative analysis of deep retinal blood perfusion, the OCTA technique for removing projection artefacts should be applied to quantify deep retinal-related blood perfusion more accurately and further subdivided middle retinal capillary plexus in NDR patients. Finally, limited by the current scope of detection and image definition of OCTA technology, this study only performed fixed-range OCTA scanning and quantitative analysis among NDR patients. In the future, wide-angle OCTA technology should be applied to evaluate the pan-retinal blood perfusion of NDR patients.

## 5 Conclusion

In conclusion, this study suggests that retinal blood flow perfusion changes and retinal nerve changes can occur in NDR patients and that there is a correlation between the two phenomena. Optical coherence tomography angiography may be a useful tool for detecting preclinical microvascular and neurological changes in NDR patients. This method can help to provide a clinical basis for future pathogenesis research of DR and diabetic retinal neurodegeneration, as well as the follow-up monitoring of retinal blood flow perfusion and retinal nerve changes in diabetic patients.

## Data availability statement

The original contributions presented in the study are included in the article/**Supplementary Material**. Further inquiries can be directed to the corresponding authors.

## Ethics statement

The studies involving human participants were reviewed and approved by the Ethics Committee of Peking University First Hospital. The patients/participants provided their written informed consent to participate in this study.

## Author contributions

Study conception and design: JCH, SJZ. Data collection: JTD, XPG. Data analysis and interpretation: YDZ, LY. Drafting of the article: All. Critical revision of the article: All. All authors contributed to the article and approved the submitted version.
