# Supplementary material for: Changes in and the association of retinal blood perfusion and retinal nerves in diabetic patients without retinopathy
Source: Front Endocrinol (Lausanne). 2023 Jan 17;13:1036735. doi: 10.3389/fendo.2022.1036735 (PMC9887038; doi:10.3389/fendo.2022.1036735)
Supplement: Supplementary file 1 [file DataSheet_1.docx]

Appendix Table 1. Basic characteristics of the subjects

| Indicators | The NDR group（n=86） | The control group（n=64） | *χ*^2^/*t value* | *P value* |
| --- | --- | --- | --- | --- |
| Male（case） | 47（54.7%） | 29（45.3%） | 1.280 | 0.258 |
| Age（year） | 59.8±9.8 | 61.8±7.7 | -1.336 | 0.184 |
| logMAR BCVA | 0.023±0.074 | 0.006±0.081 | 1.401 | 0.163 |
| Axial length（mm） | 23.51±0.91 | 23.43±0.98 | 0.519 | 0.604 |

Note: Sex composition is expressed by the number of male cases (percentage), and the *χ*^2^ test is used for the comparison between the two groups. Age, logMAR BCVA, and ocular axis length were expressed by ‾*x*±*s*, and an independent sample t-test was used for comparison between the two groups; NDR: Diabetes mellitus without diabetic retinopathy.

Appendix Table 2

Comparison of FAZ area, circumference and circularity between affected eyes in NDR group and normal control group

| FAZ parameter | NDR Group（n=86） | Normal control group（n=64） | *Z* value | *P* value |
| --- | --- | --- | --- | --- |
| area（mm^2^） | 0.331（0.268，0.413） | 0.285（0.213，0.380） | -2.757 | 0.006 |
| Perimeter（mm） | 2.555（2.298，2.813） | 2.225（1.978，2.648） | -3.618 | <0.001 |
| Circularity | 0.660（0.610，0.720） | 0.690（0.640，0.730） | -1.780 | 0.075 |

Note: measurement data are expressed by *M* (*P_25_*, *P_75_*), and Mann Whitney U test is used for comparison between the two groups; NDR: diabetes without diabetes retinopathy, FAZ: foveal avascular area.

Appendix Table 3.Comparison of VLD around the optic disc between eyes in NDR group and normal control group

| around the optic disc VLD（mm^-1^） | NDR Group（n=86） | Normal control group（n=64） | *Z* value | *P* value |
| --- | --- | --- | --- | --- |
| average | 21.05（19.18，21.70） | 19.30（19.00，19.60） | -4.383 | <0.001 |
| superior | 21.05（19.10，22.10） | 21.95（21.00，22.68） | -4.324 | <0.001 |
| temporal | 20.85（19.60，21.80） | 22.00（20.93，22.30） | -3.585 | <0.001 |
| inferior | 21.00（19.10，22.00） | 22.15（21.23，23.08） | -4.495 | <0.001 |
| nasal side | 21.20（19.40，21.90） | 22.25（20.83，23.20） | -3.963 | <0.001 |

Note: measurement data are expressed by *M* (*P_25_*, *P_75_*), and Mann Whitney U test is used for comparison between the two groups; NDR: diabetes without diabetes retinopathy; VLD: blood flow length density.

Appendix Table 4. Results of PD around the optic disc in the affected eyes of NDR group and the normal control group

| around the optic disc PD | NDR Group（n=86） | Normal control group（n=64） | *Z* value | *P* value |
| --- | --- | --- | --- | --- |
| average | 0.3828（0.3575，0.3950） | 0.3925（0.3775，0.4090） | -3.396 | 0.001 |
| superior | 0.3814（0.3508，0.4018） | 0.3990（0.3773，0.4170） | -3.899 | <0.001 |
| temporal | 0.3885（0.3630，0.4083） | 0.4010（0.3840，0.4113） | -2.539 | 0.011 |
| inferior | 0.3800（0.3580，0.3955） | 0.3995（0.3810，0.4120） | -3.842 | <0.001 |
| nasal side | 0.3815（0.3595，0.3995） | 0.3965（0.3748，0.4140） | -2.617 | 0.009 |

Note: measurement data are expressed by *M* (*P_25_*, *P_75_*), and Mann Whitney U test is used for comparison between the two groups* P < 0.05, NDR: diabetes without diabetes retinopathy; PD: perfusion density.

Appendix Table 5.Gcipl thickness in macular area of affected eyes in NDR group and normal control group

| GCIPL thickness（μm） | NDR Group（n=86） | Normal control group（n=64） | *Z* value | *P* value |
| --- | --- | --- | --- | --- |
| average | 82.2±7.7 | 85.5±5.1 | -3.202 | 0.002 |
| minimum | 77.1±8.5 | 82.4±5.5 | -4.606 | <0.001 |
| upper | 83.0±9.4 | 86.2±5.0 | -2.714 | 0.008 |
| superior temporal | 82.5±8.1 | 84.3±5.0 | -1.596 | 0.113 |
| subtemporal | 82.1±7.9 | 85.2±5.8 | -2.726 | 0.007 |
| Below | 78.7±7.7 | 83.0±5.7 | -3.744 | <0.001 |
| nasal inferior | 81.9±9.4 | 85.7±6.1 | -3.065 | 0.003 |
| nasal superior | 84.6±8.9 | 88.3±5.7 | -2.926 | 0.004 |

Note: the measurement data are expressed by $\overline{X}$± s, and the independent sample t test is used for the comparison between the two groups; NDR: diabetes without diabetes retinopathy; Gcipl: plexiform layer in ganglion cells

Appendix Table 6. RNFL thickness around the optic disc of the affected eyes in the NDR group and the normal control group

| RNFL thickness（μm） | NDR Group（n=86） | Normal control group（n=64） | *Z* value | *P* value |
| --- | --- | --- | --- | --- |
| average | 94.5±9.9 | 100.6±8.5 | -3.945 | <0.001 |
| upper | 116.2±16.4 | 123.6±13.4 | -2.970 | 0.003 |
| temporal | 70.8±12.8 | 72.4±10.4 | -0.826 | 0.410 |
| Below | 120.9±17.8 | 130.8±15.7 | -3.523 | 0.001 |
| nasal side | 70.5±8.9 | 75.0±11.2 | -2.649 | 0.009 |

Note: the measurement data are expressed by‾*x*±*s*, and the independent sample t test is used for the comparison between the two groups* P<0.05，**P<0.01 ； NDR: diabetes without diabetes retinopathy; RNFL: retinal nerve fiber layer.

Appendix Table 7. Correlation between perioptic disc blood perfusion parameters and RNFL thickness in NDR group

| Blood perfusion parameters around the optic disc | | RNFL thickness around optic disc | | | | | | | | | | | | | |
| --- | --- | --- | --- | --- | --- | --- | --- | --- | --- | --- | --- | --- | --- | --- | --- |
|  |  | average | | upper | | | | temporal | | Below | | | nasal side | | |
|  |  | *Ρ* value | *P* value | | *Ρ* value | *P* value | | *Ρ* value | *P* value | *Ρ* value | *P* value | | | *Ρ* value | *P* value |
| VLD | average | 0.090 | 0.408 | | -- | -- | -- | | -- | -- | | -- | | -- | -- |
|  | upper | -- | -- | | 0.022 | 0.838 | -- | | -- | -- | | -- | | -- | -- |
|  | temporal | -- | -- | | -- | -- | 0.133 | | 0.222 | -- | | -- | | -- | -- |
|  | Below | -- | -- | | -- | -- | -- | | -- | 0.133 | | 0.221 | | -- | -- |
|  | nasal side | -- | -- | | -- | -- | -- | | -- | -- | | -- | | 0.189 | 0.082 |
| PD | average | 0.083 | 0.445 | | -- | -- | -- | | -- | -- | | -- | | -- | -- |
|  | upper | -- | -- | | 0.003 | 0.980 | -- | | -- | -- | | -- | | -- | -- |
|  | temporal | -- | -- | | -- | -- | 0.122 | | 0.262 | -- | | -- | | -- | -- |
|  | Below | -- | -- | | -- | -- | -- | | -- | 0.134 | | 0.218 | | -- | -- |
|  | nasal side | -- | -- | | -- | -- | -- | | -- | -- | | -- | | 0.113 | 0.299 |

Note: Spearman correlation analysis is adopted; n=86； NDR: diabetes without diabetes retinopathy; RNFL: retinal nerve fiber layer; VLD: blood flow length density; PD: perfusion density; *ρ*： Spearman correlation coefficient.

**Attached figure:**

**
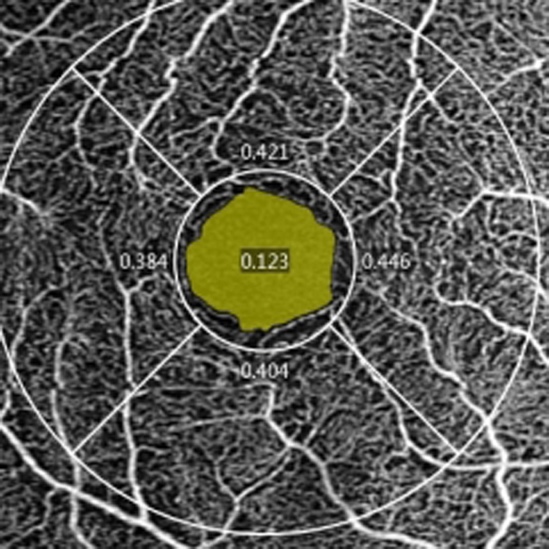
**

**Appendix Figure 1** Schematic diagram of the FAZ region automatically identified by the OCTA system software (Take the right eye as an example)

Note: The yellow area in the figure is the FAZ area. OCTA: optical coherence tomography angiography; FAZ: foveal avascular area.

**
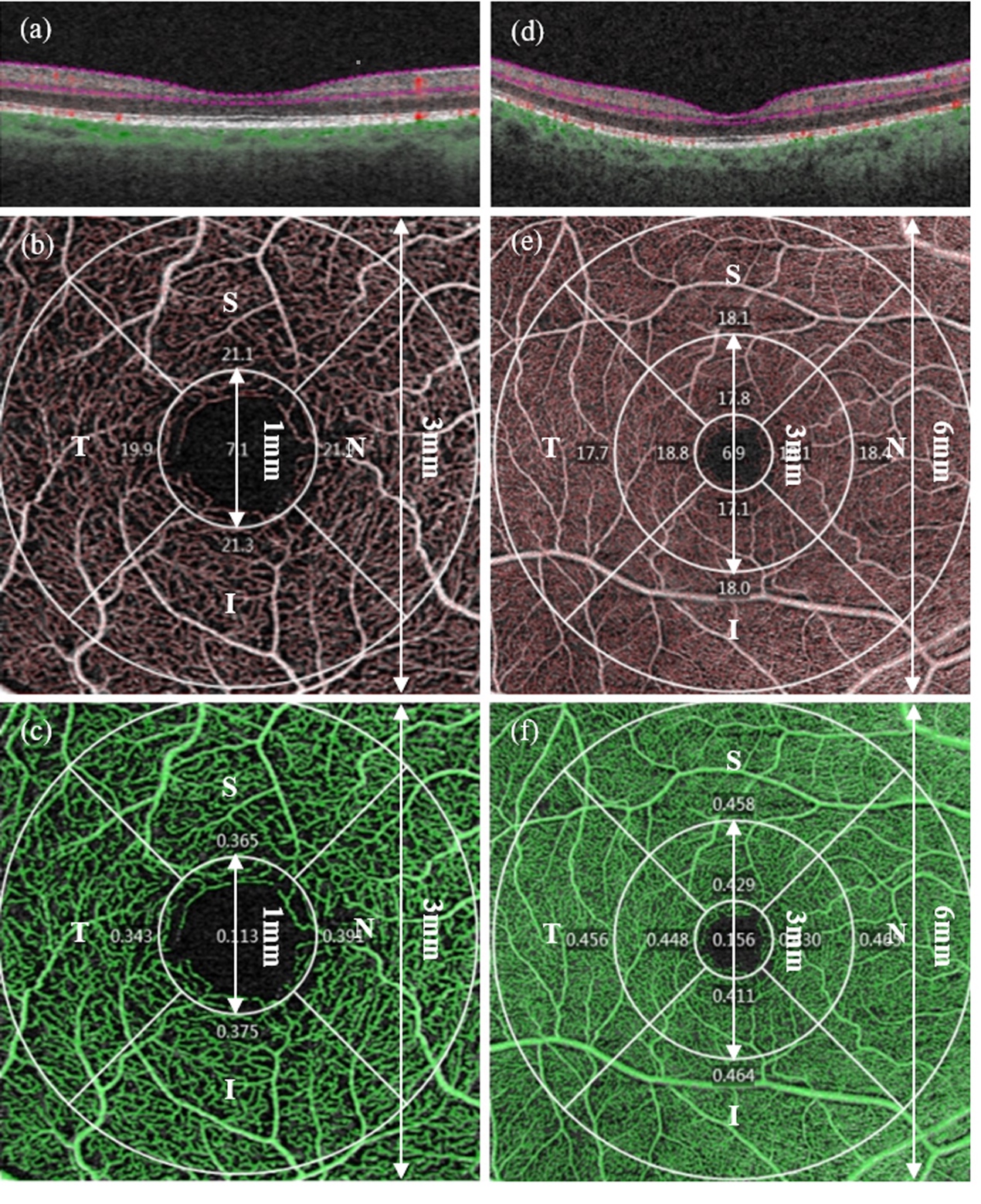
**

**Appendix Figure 2** Schematic diagram of measurement area and region of vessel perfusion parameter of OCTA in the macular area (Taking the right eye as an example)

Note: (a) the horizontal B scan image across the fovea in 3 mm×3 mm scan. The inner boundary of the superficial retina is ILM (purple dotted line above), and the outside boundary is the approximate form of IPL, equivalent to the junction of IPL and INL (purple dotted line below). (b) Display the superficial retinal en face image and the structured blood flow signal measuring the VLD automatically identified by the system software corresponding to (a) (red line). Foveal area: a circular area of 1 mm diameter centered on the macular fovea. Parafoveal area: an annular area between two concentric circles of 1 mm and 3 mm centered on the macular fovea, and is further divided into four quadrants: superior (S), temporal (T), inferior (I), and nasal (N). (c) Display the superficial retinal en face image and the blood flow signal measuring PD automatically identified by the system software corresponding to (a) (green area). Measurement region is the same as (b). (d) the horizontal B scan image across the fovea in the 6 mm×6 mm scan, with the inner and the outer boundary of the superficial retina being in common (a). (e) Display the superficial retinal en face image and the structured blood flow signal measuring the VLD automatically identified by the system software corresponding to the same (d) (red line). Perifoveal area: an annular area between two concentric circles of 3 mm and 6 mm centered on the macular fovea, and is further divided into four quadrants: S, T, I, and N. (f) Display the superficial retinal en face image and the blood flow signal measuring PD automatically identified by the system software corresponding to (d) (green area). Measurement region is the same as (e). OCTA: optical coherence tomography angiography; ILM: inner limiting membrane; IPL: internal plexiform layer; VLD: vessel length density; PD: perfusion density.

**
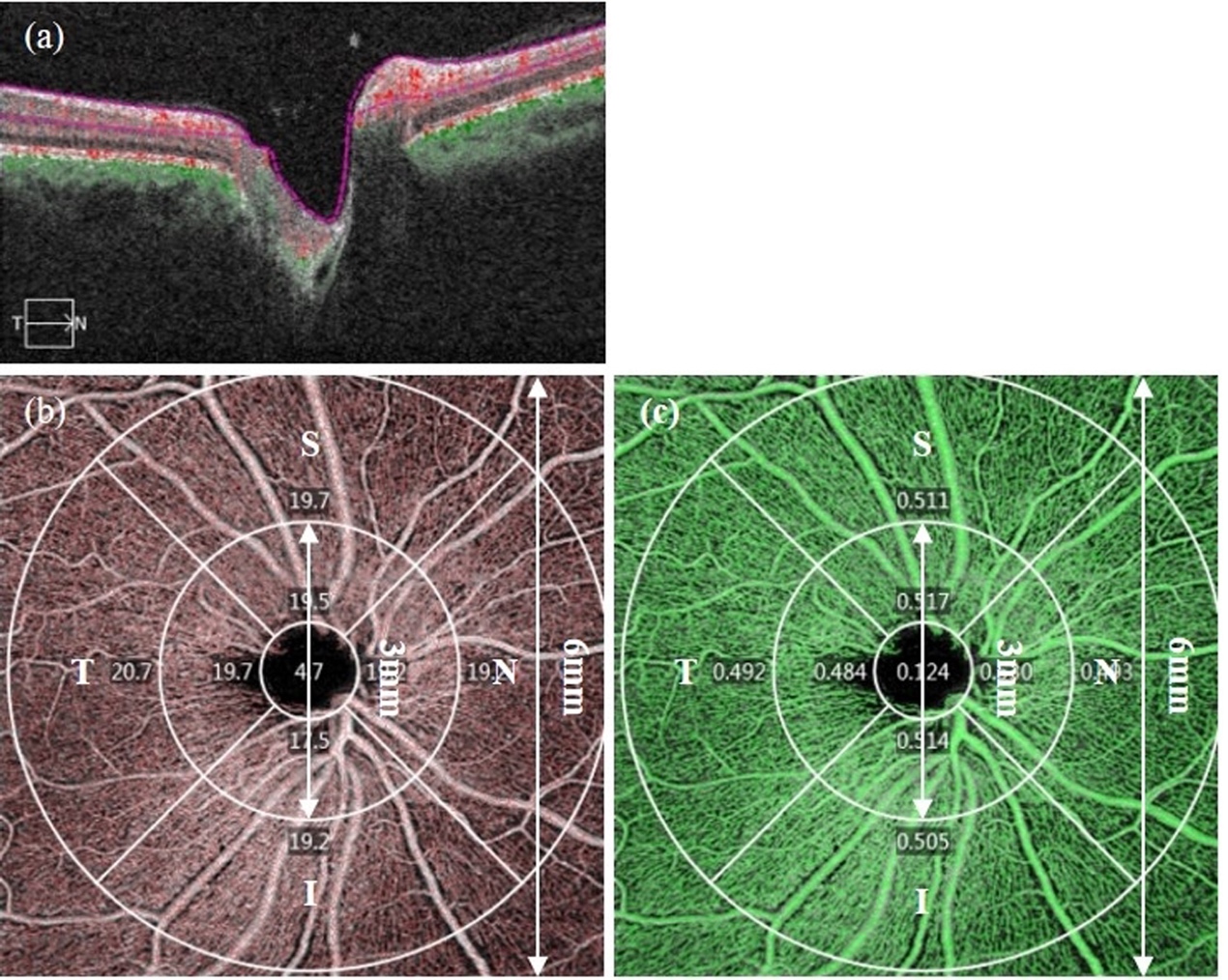
**

**Appendix Figure 3** Schematic diagram of measurement area and region of vessel perfusion parameter of OCTA around the optic disc (Taking the right eye as an example)

Note: (a) the horizontal B scan image across the center of the optic disc in 6 mm×6 mm scan. The inner boundary of the superficial retina is ILM (purple dotted line above), and the outside boundary is the approximate form of IPL, equivalent to the junction of IPL and INL (purple dotted line below). (b) Display the superficial retinal en face image and the structured blood flow signal measuring the VLD automatically identified by the system software corresponding to (a) (red line). The blood perfusion measurement area around the optic disc is the annular area between two concentric circles of 3 mm and 6 mm centered on the center of the optic disc, and is further divided into four quadrants: superior (S), temporal (T), inferior (I), and nasal (N). (c) Display the superficial retinal en face image and the blood flow signal measuring PD automatically identified by the system software corresponding to (a) (green area). Measurement region is the same as (b). OCTA: optical coherence tomography angiography; ILM: inner limiting membrane; IPL: internal plexiform layer; VLD: vessel length density; PD: perfusion density.

**
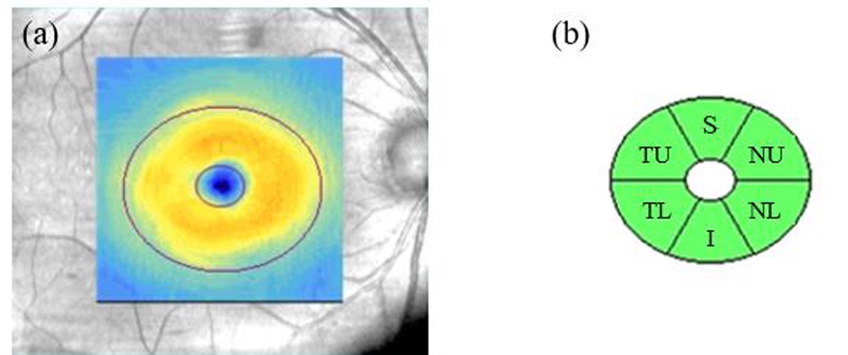
**

**Appendix Figure 4** Schematic diagram of measurement area and region of GCIPL thickness of OCTA in the macular area (Taking the right eye as an example)

Note: (a) Measurement area: an elliptical annular area of 14.13 mm^2^ (An elliptical annular region between the two purple ellipses). The horizontal and vertical diameter are 1.2 mm and l.0 mm of the inner ring, and 4.8 mm and 4.0 mm of the outer ring, respectively. (b) Measurement region: The elliptical ring area is further divided into six quadrants: superior (S), temporal upper (TU), temporal lower (TL), inferior (I), nasal lower (NL), and nasal upper (NU). OCT: optical coherence tomography; GCIPL: ganglion cell-inner plexiform layer.

**
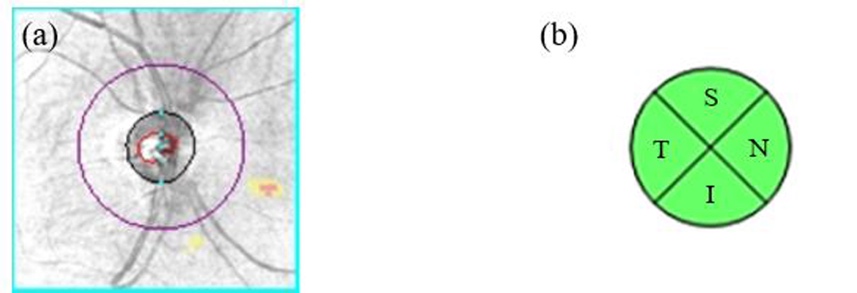
**

**Appendix Figure 5** Schematic diagram of measurement area and region of RNFL thickness of OCTA in the macular area (Taking the right eye as an example)

Note: (a) Measurement area: a circular measurement ring with 3.46 mm diameter at the center of the optic disc (purple circle). (b) Measurement region: The measurement ring is further divided into four quadrants: superior (S), temporal (T), inferior (I), and nasal (N). OCT: optical coherence tomography; RNFL: retinal nerve fiber layer.

**
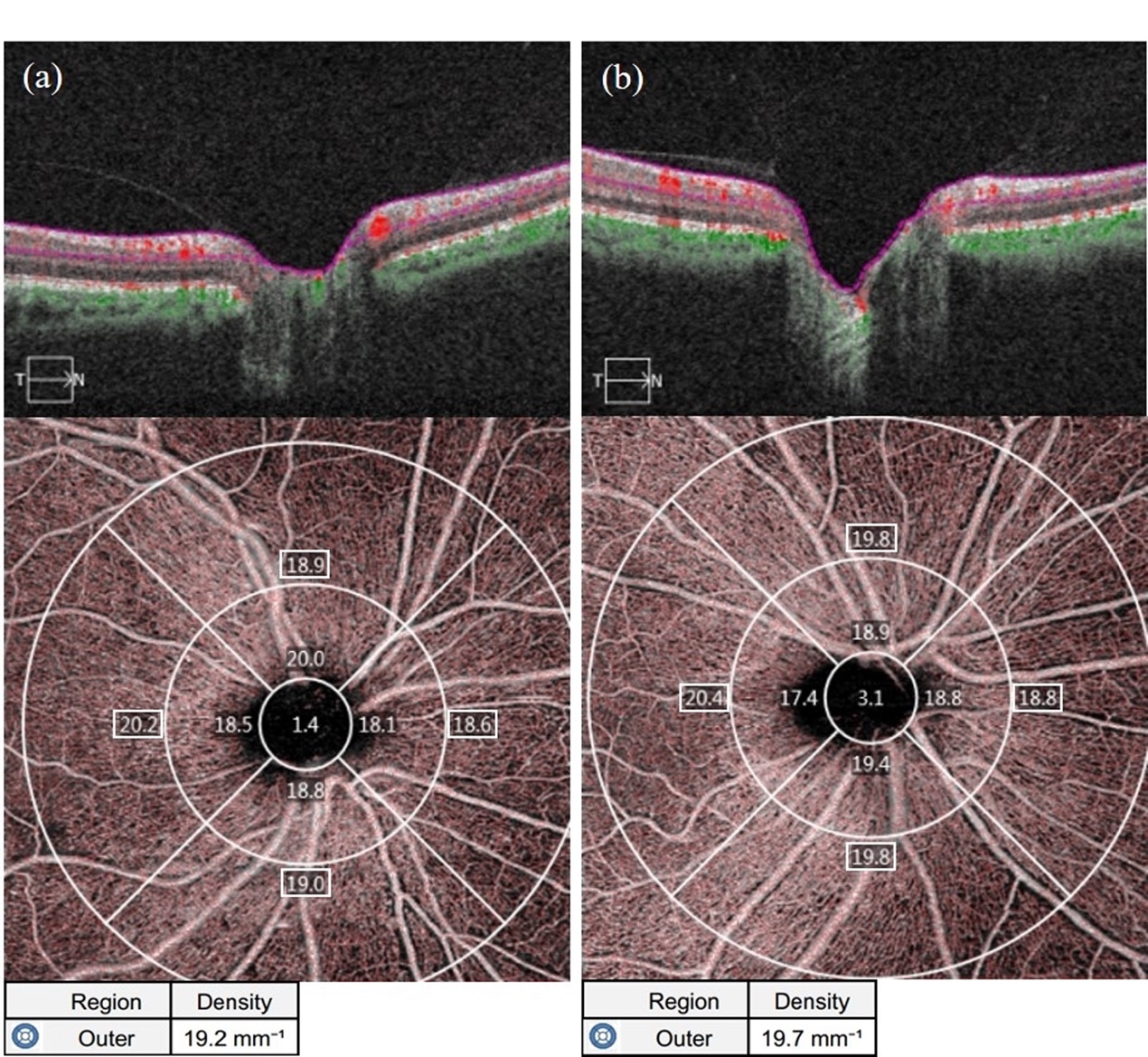
**

**Appendix Figure 6** Representative OCTA images of VLD measurements around the optic disc of the affected eyes in the NDR group and the tested eyes in the control group

Note: (a) Results of 6 mm×6 mm B scan of the OCTA, the en face image of the superficial retina and the average VLD around the optic disc (outer item in the table, the same below)and the VLD of each quadrant (The corresponding region number in the figure, the same below) automatically measured by the system software of the right eye of the 66-year-old male NDR patient. (b) Results of 6 mm×6 mm B scan of the OCTA, the en face image of the superficial retina and the average VLD around the optic disc and the VLD of each quadrant automatically measured by the system software of the right eye of the 54-year-old female NDR patient. The VLD on average around the optic disc and in each quadrant of affected eye of this NDR patient was smaller than that of the tested eyes of this control. NDR: diabetes mellitus without diabetic retinopathy; VLD: vessel length density; OCTA: optical coherence tomography angiography.

**
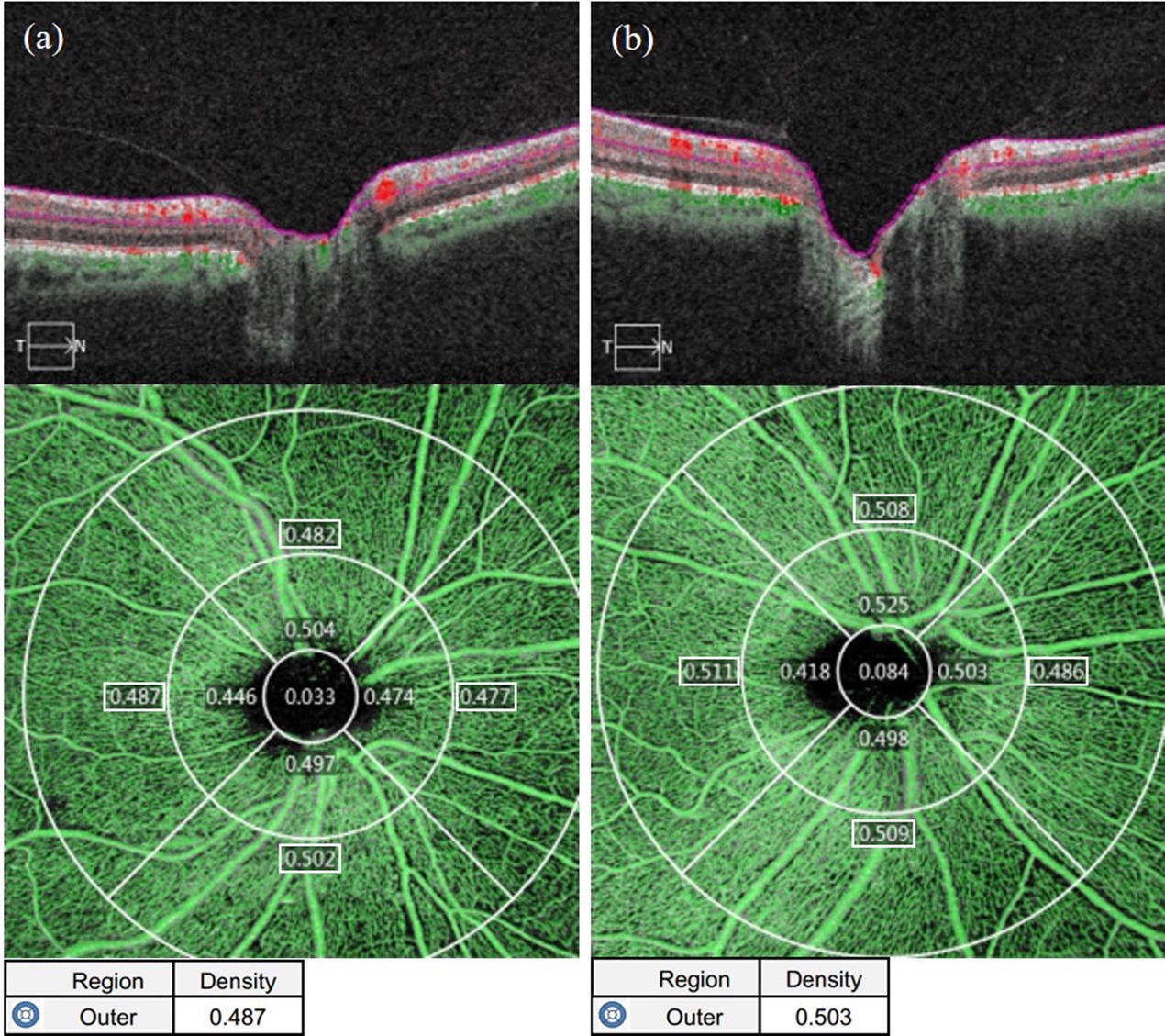
**

**Appendix Figure 7** Representative OCTA images of PD measurements around the optic disc of the affected eyes in the NDR group and the tested eyes in the control group

Note: (a) Results of 6 mm×6 mm B scan of the OCTA, the en face image of the superficial retina and the average PD around the optic disc (outer item in the table, the same below)and the PD of each quadrant (The corresponding region number in the figure, the same below) automatically measured by the system software of the right eye of the 66-year-old male NDR patient. (b) Results of 6 mm×6 mm B scan of the OCTA, the en face image of the superficial retina and the average PD around the optic disc and the PD of each quadrant automatically measured by the system software of the right eye of the 54-year-old female NDR patient. The PD on average around the optic disc and in each quadrant of affected eye of this NDR patient was smaller than that of the tested eye of this control. NDR: diabetes mellitus without diabetic retinopathy; PD: perfusion density; OCTA: optical coherence tomography angiography.
